# Dissecting Term at the University of Buffalo

**Published:** 1863-09

**Authors:** 


DISSECTING TERM AT THE UNIVERSITY OF BUFFALO.—We desire to call the attention of medical students to the time of opening of the Dissecting Rooms in the University of Buffalo. The announcement of Lectures and the Journal advertisement have both given notice incorrectly. This term commences the first Wednesday in October instead of the second, thus giving ample time for full and complete dissections previous to the commencement of the Lecture Term. The Demonstrator of Anatomy, SAMUEL W. WETMORE, M. D., will give his undivided attention to the instruction and assistance of those who attend this important preliminary course, and we have no doubt his ability and energy will make this opportunity of the greatest value to all students who would obtain a really practical knowledge of anatomy.

